# Hepcidin Expression in Iron Overload Diseases Is Variably Modulated by Circulating Factors

**DOI:** 10.1371/journal.pone.0036425

**Published:** 2012-05-07

**Authors:** Giulia Ravasi, Sara Pelucchi, Paola Trombini, Raffaella Mariani, Naohisa Tomosugi, Giulia Litta Modignani, Matteo Pozzi, Elizabeth Nemeth, Tomas Ganz, Hisao Hayashi, Donatella Barisani, Alberto Piperno

**Affiliations:** 1 Department of Clinical Medicine and Prevention, University of Milano-Bicocca, Monza, Italy; 2 Centre for Diagnosis and Treatment of Hemochromatosis, S. Gerardo Hospital, Monza, Italy; 3 Division of Nephrology, Department of Internal Medicine, Kanazawa Medical University, Ishikawa, Japan; 4 Department of Medicine and Pathology, David Geffen School of Medicine, University of California Los Angeles (UCLA), Los Angeles, California, United States of America; 5 Department of Experimental Medicine, University of Milano-Bicocca, Monza, Italy; 6 Consortium of Human Molecular Genetics, Monza, Italy; Kyushu Institute of Technology, Japan

## Abstract

Hepcidin is a regulatory hormone that plays a major role in controlling body iron homeostasis. Circulating factors (holotransferrin, cytokines, erythroid regulators) might variably contribute to hepcidin modulation in different pathological conditions. There are few studies analysing the relationship between hepcidin transcript and related protein expression profiles in humans. Our aims were: a. to measure hepcidin expression at either hepatic, serum and urinary level in three paradigmatic iron overload conditions (hemochromatosis, thalassemia and dysmetabolic iron overload syndrome) and in controls; b. to measure mRNA hepcidin expression in two different hepatic cell lines (HepG2 and Huh-7) exposed to patients and controls sera to assess whether circulating factors could influence hepcidin transcription in different pathological conditions. Our findings suggest that hepcidin assays reflect hepatic hepcidin production, but also indicate that correlation is not ideal, likely due to methodological limits and to several post-trascriptional events. *In vitro* study showed that THAL sera down-regulated, *HFE*-HH and C-NAFLD sera up-regulated hepcidin synthesis. *HAMP* mRNA expression in Huh-7 cells exposed to sera form C-Donors, *HFE*-HH and THAL reproduced, at lower level, the results observed in HepG2, suggesting the important but not critical role of HFE in hepcidin regulation.

## Introduction

Hepcidin (*606464) is a regulatory hormone that plays a major role in controlling body iron homeostasis. It is mainly synthesised in hepatocytes and secreted in the blood as a mature 25-aa peptide which binds to ferroportin (*604653), the only known cellular iron exporter, causing ferroportin internalization and degradation and, in turn, inhibition of iron absorption by enterocytes and iron release from macrophages [1], [2]. Inadequate hepcidin production relative to body iron stores results in increased iron absorption and may lead to iron overload. Hepcidin production is mainly regulated at transcriptional level by several signalling pathways [1]. Thalassemia syndromes (OMIM #613985) and other anemias with ineffective erythropoiesis are characterised by marked reduction of hepcidin synthesis caused by a still unidentified erythroid signal [3], [4]. Hereditary defects of the proteins involved in hepcidin regulatory pathways are responsible for the different forms of Hereditary Hemochromatosis (HH, OMIM#235200) [1], [5]. Among them, HH type 1 is the most common in Caucasian population and is caused by the presence of homozygous p.C282Y mutation in *HFE* (*HFE*-HH, *613609). This leads to the lack of protein expression on hepatocyte cell membrane, inability to interact with transferrin receptor (TFR) 1 (*190010) and 2 (*604720) and reduced efficiency in activating hepcidin signalling [6]. A peculiar, but very common iron overload disorder is the Dysmetabolic Iron Overload Syndrome (DIOS) which is characterized by mild to moderate iron overload in association with obesity and alterations of lipid or glucose metabolism, insulin resistance and Non Alcoholic Fatty Liver Disease (NAFLD) [7]. The mechanism of iron overload in DIOS is still undefined. Recently, it was shown that hepcidin at either mRNA [8], serum [9] or urinary [8], [9] level is increased in DIOS as compared to controls, although it was apparently inadequate compared to the amount of body iron stores. In addition, discrepancies (lack of correlation) between mRNA expression and urinary hepcidin level [8] and between serum and urinary hepcidin levels [9] have been reported in these patients, differently from what observed in patients with HH [8] and with non-HH-related hepatic cirrhosis [10]. It has been suggested that inflammatory-dependent cytokines activation, extrahepatic hepcidin production by adipose tissue as well as post-translational modifications, or abnormal interaction with ferroportin at the target sites might be involved in causing high hepcidin levels in DIOS [11]. Since the discovery of hepcidin and the demonstration of its pivotal role in iron homeostasis, there has been a great interest in measuring this hormone in tissues and biological fluids to improve our understanding of iron-related diseases, including DIOS. To our knowledge, data comparing mRNA, serum or urinary hepcidin levels in humans are scanty. So, we collected patients with three emblematic iron overload conditions, e.g. *HFE*-related HH, thalassemia major and DIOS to measure hepcidin expression at either mRNA, serum or urinary level compared to controls. Second, based on the hypothesis that circulating factors might variably contribute to hepcidin modulation in different pathological conditions, we performed *in vitro* studies to evaluate the effects of sera of patients and controls on hepcidin transcription in two different hepatic cell lines.

## Materials and Methods

### Patients

Seventy-nine patients including 23 with *HFE*-hemochromatosis (*HFE*-HH), 47 with DIOS, and 9 with b-thalassemia major (THAL) were enrolled in the study. Control groups consist of: *a.* 15 patients with non-alcoholic fatty liver disease (NAFLD) without iron overload which were taken as controls (C-NAFLD) for evaluating both hepatic mRNA and serum hepcidin levels; b. 28 healthy blood donors at their first donation which were taken as controls (C-Donors) for serum hepcidin levels and for *in vitro* study.

General exclusion criteria were: HBV or HCV infection, autoimmune hepatitis, alpha1-antitrypsin deficiency or Wilson’s disease; alcohol intake >40 g/day in men and >20 g/day in women. *HFE*-HH patients were untreated p.C282Y homozygotes with increased serum iron indices. Diagnosis of DIOS was based on the presence of hepatic iron overload with one or more component of the metabolic syndrome (according to NCEP-ATPIII criteria) [12]. In this group of patients we excluded the presence of other known causes of iron overload: history of blood transfusions or treatment with parenteral iron; HH type I and IV (OMIM#606069) by genotyping *HFE* for p.C282Y and p.H63D variants and by sequencing *SLC40A1* (Gene ID: 30061). HH type II (OMIM#613313 and #602390) and III (OMIM#604250), iron loading anemias, acute and chronic inflammatory disorders, chronic hepatic diseases, end-stage liver disease, porphyria cutanea tarda and aceruloplasminemia were excluded on clinical, biochemical and hepatic histological grounds. All DIOS patients carried the *HFE* wild-type genotype but two were heterozygous for p.H63D. Liver biopsy was performed in 67 patients (14 with *HFE*-HH, 47 with DIOS and 6 with THAL) and in 15 controls with NAFLD for diagnostic or prognostic purposes. 30 of 47 DIOS patients (64%) had NAFLD defined by the presence of at least 5% steatosis in liver biopsy [13]. The fifteen controls with NAFLD had no hepatic iron overload (Deugnier score <6) and absent or minimal fibrosis at liver biopsy.

The study, including the overall plan and the informed consent form was reviewed and approved by the institutional review boards of San Gerardo Hospital in Monza. All patients had given written informed consent to genetic testing analysis and liver biopsy before enrolment in the study according to the Ethical Committee of our Institution.

### Methods

Biochemical indices were measured at the time of diagnosis or time of liver biopsy (when performed) in *HFE*-HH, DIOS and in C-NAFLD, at the time of first donation in C-Donors, and at the time of liver biopsy in THAL. Body mass index (BMI), blood pressure and alcohol intake were also measured at diagnosis.

#### Iron and other biochemical indices

Serum iron, transferrin and ferritin, serum glucose, total cholesterol, HDL, triglycerides, alanine-aminotransferases, and γ-glutamyl transferase were measured by standard methods. Transferrin saturation was calculated as follows {serum iron (µg/dl)/[transferrin (mg/dl)x1.41]}. Serum IL-6 (*147620) was measured by ELISA assay (R&D Systems Inc. MN, USA).

#### Hepcidin in serum and urine

Urinary hepcidin concentration was assessed in 8 C-NAFLD, 28 DIOS and 15 *HFE*-HH patients. Urine were collected in the morning, fasting, just before liver biopsy, preserved with 0.05% sodium azide and stored at −80°C until measurement. Urinary hepcidin immunodot assay was performed as previously described [14]. Urinary hepcidin was normalized to urinary creatinine concentration (ng hepcidin/mg creatinine). Quantification of serum hepcidin-25 was performed in 47 DIOS, 21 *HFE*-HH and 8 THAL patients, as well as in 15 C-NAFLD and 24 C-Donors. After collection, sera were stored at −80°C until measurement. Serum hepcidin-25 levels were determined by liquid chromatography–tandem mass spectrometry (LC/MS/MS)-based assay as reported [15], [16]. Intra-assay and inter-assay coefficients of variation (CVs) were less than 6.7 and less than 8.8%, respectively. The lower limit of detection was 1.0 ng/mL with a signal to noise ratio of 10∶1 [17].

#### Liver biopsy

Immediately after the procedure, the biopsy was cut into two pieces. A 2.5 cm piece was fixed in 10% formalin (pH 7.4) for histology, whereas a 0.5–1 cm of tissue from needle liver biopsy was snap-frozen in liquid nitrogen for mRNA analysis. Hepatic sections were stained with standard and Perls’ staining for routine histology and iron deposition, respectively. Grading and staging were classified as described by Ishak et al [18], and steatosis by Kleiner’s score [13]. Liver iron overload was assessed as described by Deugnier et al [19], with Hepatocyte Iron Score (HIS) ranging from 0 to 36, Sinusoidal Iron Score (SIS) from 0 to 12, and Portal Iron Score (PIS) from 0 to 12. The sum of HIS, SIS and PIS is the Total Iron Score (TIS) which ranges from 0 to 60.

#### RNA extraction and cDNA synthesis

RNA from hepatic tissue was extracted using TRIzol (Invitrogen, Grand Island, NY, USA) according to the manufacturer’s protocol, quantified by spectrophotometry and its integrity assessed by nondenaturing agarose gel. 2 µg of total RNA was used as a template for reverse transcription, performed using the High Capacity cDNA Archive kit (Applera, Foster City, CA, USA), according to the manufacturer’s protocol.

#### Real-time quantitative-PCR

mRNA expression levels of *HAMP* (Gene ID:57817) were evaluated by quantitative real time PCR (qRT-PCR); *HPRT1* (Gene ID:3251) was chosen as housekeeping gene. The analysis were performed on an ABI 7900HT (Applera, Foster City, CA, USA) using the Assays-on-Demand Gene Expression Products (Applera) according to the manufacture’s protocol (catalog no. of TaqMan Assay® are Hs99999909_m1 and Hs00221783_m1 for *HPRT1* and *HAMP*, respectively). Instrument was set up with default thermal cycler protocol provided by the producer: 50°C for 2 min, 95°C for 10 min, 95°C for 15sec and 60°C for 1 min for 40 cycles. TaqMan® Assays (20X) contain 18 µM of each primers and 5 µM for the probe. Amplification efficiencies were calculated according to our experimental conditions and were found to be 96% for *HAMP* and 98% for *HPRT1*, data in accordance with the TaqMan® Gene Expression Assays datasheet that reports a PCR efficiency of 100% (±10%). Inter-assay plate variation showed a % Coefficient of Variation (CV) of 1.17% and 1.21% for hepatic *HAMP* and *HPRT1,* respectively, in three different liver samples; %CV for cellular *HAMP* was 0.80% and 1.9% for cellular *HPRT1* in three different HepG2 and Huh-7 samples. For each PCR reaction, 15 ng of cDNA were used as a template. All analyses were carried out in triplicate; results showing a discrepancy greater than 0.3 cycle between the samples were excluded. Relative quantities present in each sample were assessed using the 2^−(ΔΔCt)^ method [20]; a cDNA generated from total liver RNA (Invitrogen, Grand Island, NY, USA) was used as external control. Non-retrotranscribed RNAs were included in each amplification plate, and the analysis regarded as valid if the fluorescence intensity in the no-template control was zero.

#### *In vitro* study

The human hepatoma HepG2 cell-line was grown in MEM (Minimum Essential Medium) supplemented with 10% heat-inactivated fetal bovin serum (FBS), glutamine and combined antibiotics, 37°C e 5% CO_2_. The human hepatoma Huh-7 cell-line was grown in DMEM (Dulbecco Modified Essential Medium) in the same conditions. For the examination of serum effect, cells were seeded in 6-well plates (300.000 cell/well). After 24 h they were starved of FBS for 24 h, after which the medium was changed to medium containing 10% human serum [21]. After an additional 48 h, the cells were harvested for RNA isolation and gene expression analysis as reported above. We treated HepG2 cells with sera of 13 *HFE*-HH, 28 DIOS, 9 THAL patients and 13 C-NAFLD and 28 C-Donors. Huh-7 were incubated with sera of 13 *HFE*-HH, 9 THAL patients and 28 C-Donors. Cell lines were bought from I.Z.S.L.E.R. (Istituto Zooprofilattico Sperimentale della Lombardia e dell'Emilia- Romagna, Brescia, Italy).

#### Statistical analysis

Data were expressed as median and range. All comparisons involving quantitative variables were performed by nonparametric tests: Kruskal-Wallis with Dunn’s test for multiple comparisons, and Mann–Whitney to compare two groups of patients. The degree of linear association between two variables was assessed by Spearman’s test. All tests were two sided and with a significance level of α equal to 0.05. Analyses were carried out by the GRAPHPAD PRISM statistical analysis software (version 3.02) (GraphPad Software, Inc., La Jolla, CA, USA).

## Results

Subjects characteristics are shown in Table 1. Excluding THAL patients, age, total cholesterol, γGT, serum glucose and hemoglobin concentration did not differ among groups.

**Table 1:** Characteristics of the subjects.

	C-Donors (n = 28)	C-NAFLD (n = 15)	DIOS (n = 47)	*HFE*-HH (n = 23)	THAL (n = 9)
**Age (yrs)**	45 (19–60)	42 (21–66)	55 (30–68)	41 (21–68)	30 (27–37)
**BMI (Kg/m^2^)**	24.1 (21–27.5)	26.3 (23–39.7)	25.4 (22–33.6)	24.2 (18.8–28)	23.9 (18.8–27.8)
**Alcohol (g/day)**	5 (0–30)	0 (0–40)	7.5 (0–30)	10 (0–60)	5 (0–40)
**Hemoglobin (gr/dL)**	14.3 (13.5–16)	15.0 (13.3–16.9)	14.2 (11.2–16.3)	14.6 (11.2–16.8)	9.8 (8.3–10.7)
**Transferrin Saturation (%)**	29 (20–41)	32.5 (21–222)	38 (9–65)	87 (3.2–98)	97 (94–106)
**Ferritin (µg/L)**	71 (53–308)	241 (41–806)	872 (351–2293)	1392 (201–4728)	1060 (520–2481)
**TIS**	ND	0 (0–6)	17 (12–29)	32* (22–38)	27.5° (16–37)
**Glucose (mg/dL)**	92 (64–101)	101 (73–203)	95 (75–146)	96 (65–131)	111 (93–189)
**Cholesterol (mg/dL)**	199 (137–266)	180 (112–227)	197.5 (125–273)	198 (145–256)	114 (62–166)
**Triglycerides (mg/dL)**	80 (42–133)	109 (34–233)	119.5 (61–505)	93 (57–180)	82 (45–346)
**Steatosis (%)**	ND	60 (0–98)	65 (0–98)	0 (0–70)	0 (0–0)
**ALT (IU/L)**	21.5 (14–38)	60 (33–103)	39 (17–118)	32.5 (12–67)	34 (12–95)
**γGT (IU/L)**	ND	51 (16–109)	39.5 (16–271)	30 (11–350)	20.5 (14–57)

Values are represented as median (range).

ND: not done; ALT: alanine aminotransferase; γ-GT: γ-glutamyl-transferase; TIS: Total Iron Score; *: available in 14 patients; °: available in 6 patients.

**Age**: DIOS vs THAL (p<0.01);

**BMI**: C-NAFLD vs C-Donors, *HFE*-HH, THAL (p<0.05);

**Hemoglobin**: THAL vs all (p<0.01);

**Transferrin saturation**: C-Donors vs *HFE*-HH, THAL (p<0.01); C-NAFLD vs *HFE*-HH, THAL (p<0.01); DIOS vs *HFE*-HH, THAL (p<0.01);

**Ferritin**: C-NAFLD vs DIOS, *HFE*-HH, THAL (p<0.01);

**TIS**: C-NAFLD vs all (p<0.01); DIOS vs *HFE*-HH (p<0.01);

**Cholesterol**: THAL vs C-Donors, DIOS, *HFE*-HH (P<0.05);

**Tryglicerides**: C-Donors vs DIOS (p<0.01);

**Steatosis**: C-NAFLD vs *HFE*-HH, THAL (p<0.01); DIOS vs *HFE*-HH, THAL (p<0.01);

**ALT**: C-Donors vs NAFLD, DIOS (p<0.01); C-NAFLD vs *HFE*-HH (p<0.05).

Iron and metabolic indices significantly differed among groups. As expected, *HFE*-HH and THAL had more severe iron overload than other groups, whereas DIOS and C-NAFLD had more metabolic abnormalities and steatosis than *HFE*-HH and THAL. To evaluate the possible effect of inflammatory activation on hepcidin levels in patients with dysmetabolic alterations, we measured IL-6 concentrations in DIOS and C-NAFLD compared to C-Donors. Serum IL-6 was higher in C-NAFLD [0.99 (0.16–21.35) pg/ml] than in DIOS [0.10 (0.10–7.010) pg/ml] and C-Donors [0.10 (0.10–4.52) pg/ml] (p<0.001).

### mRNA, Serum and Urinary Hepcidin

*HFE*-HH and THAL had the lowest mRNA, serum and urinary hepcidin among groups, whereas DIOS had the highest (Table 2). The correction of hepcidin values for the amount of storage iron (serum ferritin and TIS) underscores the inadequate production of hepcidin in *HFE*-HH and THAL, and the higher values in C-NAFLD and DIOS in the order (Table 3).

**Table 2:** Levels of *HAMP* mRNA, serum and urinary hepcidin in controls (NAFLD patients without iron overload), DIOS and *HFE*-HH patients.

	C-Donors	C-NAFLD	DIOS	*HFE*-HH	THAL
***HAMP*** ** mRNA (2^−ΔΔCt^)**	ND	(N = 15) 32.9 (3.6–52)	(N = 47) 44.6 (12.6–187.3)	(N = 14) 18.5 (3.9–56.8)	(N = 6) 13.8 (3.9–41.8)
**S-Hepcidin (ng/mL)**	(N = 24) 14 (3.3–36.5)	(N = 15) 11.1 (0.5–25.5)	(N = 47) 17.4 (2.1–65.0)	(N = 21) 4.8 (1.0–31.3)	(N = 8) 6.15 (2.3–38.5)
**U-Hepcidin (ng/mg creatinine)**	ND	(N = 8) 82 (30–156)	(N = 28) 163 (72–449)	(N = 15) 24 (2–169)	ND

Values are represented as median (range).

ND: not done.

***HAMP*** **mRNA**: DIOS vs *HFE*-HH, THAL (p<0.01);

**S-Hepcidin**: *HFE*-HH vs C-Donors, DIOS (p<0.01); NAFLD vs DIOS (p<0.05);

**U-Hepcidin**: DIOS vs *HFE*-HH (p<0.01), NAFLD (p<0.05).

**Table 3:** Ratios of *HAMP* mRNA, serum and urinary hepcidin in controls (NAFLD patients without iron overload), DIOS and *HFE*-HH patients.

	C-Donors	C-NAFLD	DIOS	*HFE*-HH	THAL
**Normalized ** ***HAMP*** ** mRNA**	**ND**	**N = 15**	**N = 47**	**N = 14**	**N = 6**
HAMP/SF	ND	0.11 (0.02–1.17)	0.05 (0.01–0.16)	0.01 (0.00–0.05)	0.01 (0.00–0.03)
HAMP/TIS	ND	8 (5.1–47.2)	3.01 (0.6–6.9)	0.43 (0.1–1.9)	0.53 (0.2–1.5)
**Normalized S-hepcidin**	**N = 24**	**N = 15**	**N = 47**	**N = 21**	**N = 8**
S-hepcidin/SF	0.11 (0.06–0.25)	0.03 (0.00–0.27)	0.02 (0.00–0.05)	0.00 (0.00–0.02)	0.00 (0.00–0.01)
S-hepcidin/TIS	ND	2.48 (1.5–12.8)	1.07 (0.15–2.9)	0.16 (0.05–0.9)	0.27 (0.14–0.9)
**Normalized** **U-hepcidin**	**ND**	**N = 8**	**N = 28**	**N = 15**	**ND**
U-hepcidin/SF	ND	0.60 (0.08–1.51)	0.19 (0.05–0.72)	0.05 (0.00–0.10)	ND
U-hepcidin/TIS	ND	40.5 (5.0–76.0)	10 (4.5–36.2)	2.12 (0.00–7.3)	ND

Values are represented as median (range).

ND: not done; SF: serum ferritin; TIS: Total Iron Score.

**HAMP/SF**: NAFLD vs *HFE*-HH, THAL (p<0.01) DIOS (p<0.05); DIOS vs *HFE*-HH (p<0.01), THAL (p<0.05);

**HAMP/TIS**: C-NAFLD vs *HFE*-HH, THAL (p<0.01); DIOS vs *HFE*-HH, THAL (p<0.01);

**S-hepcidin/SF**: C-Donors vs DIOS, *HFE*-HH, THAL (p<0.01); NAFLD vs *HFE*-HH (p<0.01), THAL (p<0.05); DIOS vs *HFE*-HH (p<0.01);

**S-hepcidin/TIS**: NAFLD vs *HFE*-HH, THAL (p<0.01); DIOS vs *HFE*-HH (p<0.01);

**U-hepcidin/SF**: *HFE*-HH vs DIOS, NAFLD (p<0.01);

**U-hepcidin/TIS**: DIOS vs *HFE*-HH (p<0.01).

### Correlation between Hepcidin Levels and Other Variables

Serum and urinary hepcidin showed a significant correlation in the whole population (r:0.62, p<0.0001) and in *HFE*-HH patients (r:0.54, p = 0.037). In the whole population, *HAMP* mRNA level correlated with serum and with urinary hepcidin concentration (r:0.32, p = 0.0057 and r:0.34, p = 0.025 respectively). Serum hepcidin also inversely correlated with transferrin saturation (r:-0.25, p = 0.014) in the whole population, but not in single groups. In the whole population (r:0.241, p = 0.013) there was a direct correlation between serum ferritin and serum hepcidin concentration, as also observed in the DIOS (r:0.38, p = 0.0082), C-Donors (r:0.837, p<0.0001) and C-NAFLD (r:0.552, p = 0.033) groups.

### *HAMP* mRNA Response to Patient’s Sera in HepG2 and Huh-7 Cell Lines

Differently to HepG2, Huh-7 carries a mutation in *HFE* leading to the lack of HFE expression on the cell membrane [22], thus being a good *in vitro* model for testing whether the absence of this protein causes a different regulation of hepcidin expression after incubation with human serum. As reported in Table 4, treatment of HepG2 cells with the sera of C-NAFLD and *HFE*-HH increased hepcidin mRNA synthesis whereas exposure to THAL sera induced a strong inhibition. By contrast, treatment of HepG2 cells with DIOS sera did not induce significant differences in hepcidin expression compared to C-Donors. Similar results were obtained in Huh-7 exposed to C-Donors, *HFE*-HH and THAL sera, although *HAMP* expression was significantly lower than in HepG2 cells in each group (Table 4). *HAMP* mRNA levels in HepG2 and Huh-7 significantly correlated (r:0.625, p<0.0001) when the whole population was considered.

**Table 4:** *HAMP* mRNA expression in HepG2 and Huh-7 cells treated with different patients sera.

	C-Donors (n = 28)	C-NAFLD (n = 13)	DIOS (n = 28)	*HFE*-HH (n = 13)	THAL (n = 9)
**HepG2 ** ***HAMP*** ** mRNA (2^−ΔΔCt^)**	2.06 (0.6–4.8)	6.34 (0.6–16.5)	2.32 (0.3–6.7)	4.13 (1.5–12.5)	0.44 (0.1–1.4)
**Huh-7 ** ***HAMP*** ** mRNA (2^−ΔΔCt^)**	1.25 (0.3–2.2)	ND	ND	2.34 (0.6–4.1)	0.12 (0.03–0.5)
**p (HepG2 vs Huh-7)**	0.003	–	–	0.022	0.005

Results are expressed as 2^−ΔΔCt^, as median (range).

ND: not done.

HepG2 *HAMP* mRNA: THAL vs all (p<0.01); *HFE*-HH vs C-Donors (p<0.05); C-NAFLD vs C-Donors (p<0.01); C-NAFLD vs DIOS (p<0.02);

Huh-7 *HAMP* mRNA: THAL vs all (p<0.01); *HFE*-HH vs C-Donors (p<0.01).

## Discussion

Our study provides some new and some confirmatory findings. First, quantification of hepcidin indicated that the amount of protein in serum and urine significantly but slightly correlated with the liver transcript in the whole population. Second, among patients with iron overload, hepcidin expression was up-regulated in DIOS and patients with NAFLD without iron overload (C-NAFLD) and down-regulated in *HFE*-HH and THAL. Third, incubation of hepatic cell lines with patients’ sera showed that circulating factors can variably modulate hepcidin expression.

There are few studies analysing the relationship between hepcidin transcript and related protein expression profiles in humans. Detivaud et al [10] and Kattamis et al [23] found a moderate correlation between urinary hepcidin and mRNA transcript in patients with chronic liver disease and thalassemia, but such correlation was not found in DIOS patients [8]. When patients with chronic hepatitis C were evaluated, Tsochatzis et al [24] found no correlation whereas Fujita et al [25] showed a nice correlation between *HAMP* hepatic transcript and serum hepcidin. The results reported in the present manuscript extend the observation to a large number of patients with different hepatic disorders. Overall, these findings suggest that hepcidin assays reflect hepatic hepcidin production, but also indicate that this correlation is not ideal. This fact is possibly due to limits in quantitative accuracy of hepcidin mRNA and protein measurements, but also to several post-trascriptional events (protein degradation and secretion, hepcidin-ferroportin interaction and hepcidin internalization at its target sites, and extra-hepatic production) which might have a role in determining the net amount of circulating and functional protein. Although hepcidin is mainly expressed in hepatocytes, it is also expressed in other organs including heart, lung, adipose and macrophage-rich tissues, whose contribution to the amount of circulating hepcidin is unknown and might vary in different pathological conditions [1].

Second, our results confirm that DIOS patients retain the ability to increase hepcidin production in response to iron load, differently to *HFE*-HH and THAL. It cannot be excluded that mild inflammation [shown by the slightly higher IL-6 levels than in healthy controls] might contribute to the increased hepcidin production. However, we previously demonstrated that iron depletion normalized hepcidin levels in DIOS, indicating that iron stores were major determinants of hepcidin increase [26]. This also explains some of the phenotypic characteristics of the syndrome: normal transferrin saturation, increased iron accumulation in macrophages and absence of progression of iron overload [9], [27], [28]. These results are even more evident when considering the ratios between hepcidin and ferritin or TIS at both the mRNA and protein levels. The correction of hepcidin data according to serum ferritin level is supposed to better assess the adequateness of hepcidin production to the stimulus induced by iron overload because of the strong correlation between hepcidin and ferritin concentrations [29]. However, this ratio should be taken with caution when considering disorders characterized by serum ferritin increase disproportionate to the amount of iron overload such as C-NAFLD and DIOS [30], [31], due to hepatocellular necrosis and local inflammation. Taking TIS as a measure of hepatic iron overload, we showed that C-NAFLD had the highest ratio among groups at each level (serum, urinary and mRNA), from 2.5 to 4 times higher than DIOS, and that DIOS had 5 or more times higher ratios than *HFE*-HH and THAL. The higher serum IL-6 in C-NAFLD likely explain the higher hepcidin/TIS ratio compared to DIOS patients.

Hepcidin transcription is modulated by different stimuli, which act as positive or negative regulators. There are four main active regulatory pathways (erythroid-, inflammatory-, iron- and hypoxia-mediated) which control hepcidin production through different signalling pathways [1], [2], [4]. Some of them are activated by factors circulating in blood: a. holotransferrin stimulates hepcidin synthesis interacting with the iron sensor complex (formed by TFR1 and 2, and HFE) at hepatocyte membrane; b. IL-6 induces hepcidin synthesis through IL-6 receptor (Gene ID: 3570) and STAT3 (Gene ID: 6764) signalling; c. factor(s) released by erythroblasts likely regulate the transcription of hepcidin in the liver according to erythropoiesis activation [32]. We then analysed modulation of *HAMP* mRNA expression induced by incubating two different hepatic cell lines (HepG2 and Huh-7) with patients’ sera. Our results indicate that: a. sera of patients with THAL down-regulated hepcidin synthesis as previously reported [33]; b. *HFE*-HH and C-NAFLD sera induced *HAMP* transcription; c. serum of patients with DIOS did not lead to significant changes as compared to C-Donors; d. *HAMP* mRNA expression in Huh-7 cells exposed to sera form C-Donors, *HFE*-HH and THAL reproduced the results observed with HepG2, but at lower level. These results allow the following conclusions to be made. First, hepcidin down-regulation induced by THAL sera confirms the existence of regulating factor(s) released by erythroblasts into the plasma to modulate hepcidin production [21], [34]. It was suggested a role for GDF15 (*605312) and TWSG1 (*605049) erythropoietic growth factors [35], but recent studies challenged the hypothesis, suggesting that other erythroid factors might be implicated [36], [37]. Hepcidin down-regulation occurred in HepG2 cells treated with THAL sera despite their high transferrin saturation which was supposed to increase hepcidin synthesis. This indicates that the hepcidin regulatory pathway triggered by the putative erythroid factor(s) predominates on the signalling cascade activated by diferric holotransferrin. The latter likely explains the increased hepcidin transcription observed when HepG2 cells, which have a normal HFE, were incubated with the iron overloaded serum of *HFE*-HH patients. DIOS sera did not induce any differences in hepcidin production as compared to C-Donors. This finding suggests that the slight increase of IL-6 level was not sufficient to induce hepcidin expression *in vitro* and further confirms that the increased mRNA and protein hepcidin levels observed in patients with DIOS mainly depend to the increased liver iron storage. By contrast, exposure of HepG2 cells with the serum of C-NAFLD patients led to the highest up-regulation of hepcidin mRNA among groups analyzed. Circulating inflammatory cytokines, as shown by the higher IL-6 levels compared to that observed in DIOS and C-Donors, were likely major determinants of hepcidin up-regulation in HepG2 cells induced by C-NAFLD sera. The results observed in Huh-7 cell lines support the role of HFE as hepatocyte iron sensor in modulating hepcidin synthesis through interaction with diferric holotransferrin, TFR−1 and −2 [6] in healthy controls, *HFE*-HH and THAL patients. Nevertheless, hepcidin response of Huh-7 cell lines to *HFE*-HH sera suggests that HFE has an important but not critical role in regulating *HAMP* transcription and that other components of the membrane iron sensor complex may activate hepcidin signalling even in the absence of HFE. This might explain the low penetrance of the p.C282Y homozygous genotype and support the idea that full expression of HH type 1 requires additional factors either genetic or acquired.
